# Balancing oncologic risk and fertility potential: a single-center study on Turner syndrome patients with Y chromosome material

**DOI:** 10.3389/fendo.2026.1793595

**Published:** 2026-05-05

**Authors:** Xia Shuai, Zhibing Guo, Dongguang Zhang, Hui Huang, Ka Chen, Yu Yang, Qinghua Hu

**Affiliations:** 1 Jiangxi Provincial Key Laboratory of Child Development and Genetics, Jiangxi Provincial Children’s Hospital (The Affiliated Children’s Hospital of Nanchang Medical College), Nanchang, China; 2 Department of Endocrinology, Metabolism and Genetics, Jiangxi Provincial Children’s Hospital (The Affiliated Children’s Hospital of Nanchang Medical College), Nanchang, China; 3 Central Laboratory, Jiangxi Provincial Children’s Hospital (The Affiliated Children’s Hospital of Nanchang Medical College), Nanchang, China

**Keywords:** anti-Müllerian hormone, fertility preservation, gonadoblastoma, ovarian reserve, Turner syndrome, Y chromosome material

## Abstract

**Introduction:**

Turner Syndrome (TS) is a chromosomal disorder characterized by partial or complete monosomy of the X chromosome. Approximately 5–10% of patients with TS harbor Y chromosome material, which is associated with an increased risk of gonadoblastoma and other gonadal malignancies. However, the indications and optimal timing of prophylactic gonadectomy remain controversial, particularly in asymptomatic individuals, because a subset of TS patients with Y chromosome material may retain residual gonadal function and fertility potential. This study aimed to determine the prevalence of Y chromosome material in TS patients, evaluate the associated risk of gonadal tumors, and assess fertility potential using molecular genetic techniques, thereby providing evidence to inform individualized clinical decision-making.

**Methods:**

A total of 224 patients with TS diagnosed between 2016 and 2024 were enrolled. Molecular genetic techniques were utilized to identify Y-sequences. In Y-positive cases, we systematically analyzed gonadal tumor incidence via histopathology, endocrine profiles (FSH, LH, AMH), and pelvic imaging findings.

**Results:**

Y-chromosome material was identified in 11.6% (26/224) of patients. Among the 16 who underwent surgery, gonadal tumors were pathologically confirmed in 4 cases (25.0%), including a tumor in a patient with cryptic Y-material undetected by karyotyping. Importantly, preoperative pelvic ultrasonography failed to detect any definite masses in these tumor-positive cases. Endocrine profiling revealed an age-dependent decline in gonadal function: while prepubertal patients maintained uncertain endocrine activity, those in or beyond puberty exhibited hypergonadotropic hypogonadism. AMH levels indicated that the window for fertility potential is narrow and typically closes by early adolescence.

**Conclusion:**

TS patients harboring Y-chromosome material face a significant risk of malignancy that cannot be reliably excluded by imaging alone. Our findings reinforce the necessity of early prophylactic gonadectomy. While individualized management may consider fertility preservation in select prepubertal patients, the rapid depletion of ovarian reserve necessitates proactive clinical decision-making.

## Introduction

Turner syndrome (TS) is a common chromosomal disorder affecting approximately 1 in 2,500 live-born females and is characterized by complete or partial monosomy of the X chromosome. Conventional cytogenetic analyses detect Y chromosome material in approximately 5% of patients with TS. However, with the application of more sensitive molecular techniques—such as fluorescence *in situ* hybridization (FISH) and polymerase chain reaction (PCR)—the prevalence of Y chromosome material has been reported to increase to 8–12% (1–7). The presence of Y chromosome sequences in TS patients (TS+Y) is clinically significant because it confers an elevated risk of gonadoblastoma, a premalignant germ cell tumor with the potential for malignant transformation. Reported incidences of gonadoblastoma in TS+Y patients range widely from 3% to 15% (8–13), depending heavily on the sensitivity of detection methods and the age at surgery. Consequently, prophylactic gonadectomy remains the long-standing clinical recommendation upon the identification of Y-chromosome material. Nevertheless, emerging evidence indicates that not all TS+Y patients uniformly lack gonadal function. Case reports and cohort studies have documented spontaneous breast development, menarche, and even natural pregnancies in a small subset of individuals with TS+Y (14, 15), suggesting that residual germ cells and endocrine activity may persist in selected patients. These observations highlight the clinical dilemma of balancing oncologic safety against the potential preservation of gonadal function and future fertility.

Recent clinical practice guidelines from the European Society of Endocrinology advocate for an individualized management strategy in TS+Y patients (16), emphasizing shared decision-making regarding the timing of prophylactic gonadectomy. This approach necessitates a rigorous assessment of the risks of gonadoblastoma and dysgerminoma against the potential benefits of preserving endogenous hormone production and fertility. However, existing literature often focuses disproportionately on either oncologic risk or reproductive outcomes, and large-scale multicenter analyses are frequently constrained by heterogeneity and statistical bias. Furthermore, comprehensive data on fertility-related biomarkers—specifically anti-Müllerian hormone (AMH)—in the TS+Y subpopulation remain sparse (17, 18). In this context, the present single-center study aims to systematically evaluate the prevalence of both overt and cryptic Y-chromosome material in patients with Turner syndrome using advanced molecular genetic techniques. We further compare the risk of gonadal malignancy between patients with cytogenetically visible Y chromosomes and those with PCR-detected cryptic fragments. Additionally, we analyze clinical parameters including spontaneous pubertal development, menstrual status, pelvic ultrasonographic findings, and serum levels of follicle-stimulating hormone (FSH), luteinizing hormone (LH), and AMH. By integrating oncologic, endocrine, and imaging data, this study seeks to clarify the clinical equilibrium between prophylactic surgery and fertility preservation, thereby providing robust evidence to support individualized management strategies for TS+Y patients. Notably, our findings emphasize the clinical significance of cryptic Y-chromosome material, which may confer a comparable tumor risk to visible Y fragments.

## Methods

### Study design and patient population

**Figure 1:**
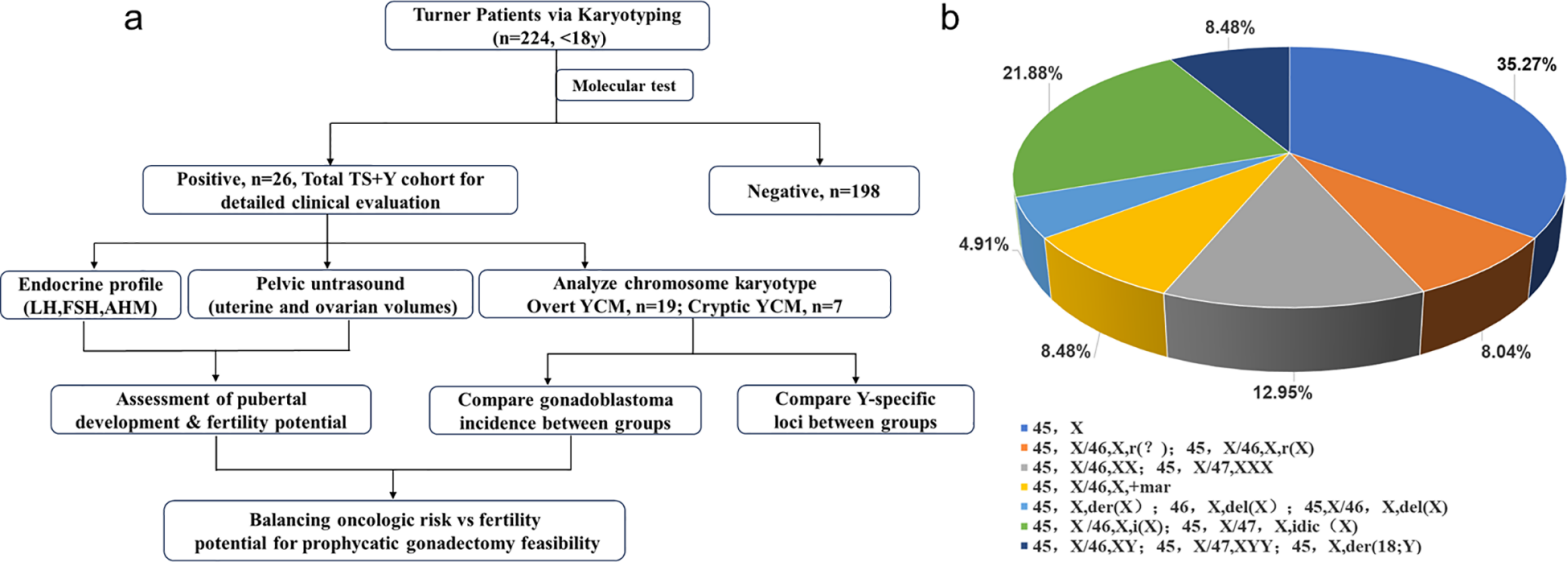
**(a)** Flowchart of patient screening and clinical evaluation. A total of 224 TS patients were screened, identifying 26 cases with Y-chromosome material (YCM). The diagram outlines the multi-dimensional assessment (genetic, endocrine, and imaging) used to evaluate oncologic risk and fertility potential. **(b)** The percentage of each chromosomal karyotype in 224 TS patients.

This study included 224 girls diagnosed with Turner syndrome who were evaluated at a tertiary pediatric medical center between January 2016 and May 2024. The patient recruitment and screening process are summarized in the study flow diagram (**Figure 1a**). The diagnosis of TS was confirmed in all patients by peripheral blood karyotype analysis using standard cytogenetic techniques. For each patient, a minimum of 30 metaphases were routinely analyzed. In cases where mosaicism was detected or clinically suspected, the analysis was extended to 50–100 metaphases to ensure the detection of low-level cell lines. All enrolled patients were younger than 18 years at the time of diagnosis. Clinical data were extracted from medical records and included age at diagnosis, presenting clinical features, and karyotype results. The study protocol was approved by the Institutional Ethics Committee of a tertiary pediatric medical center (Ethical Review No. JXSETYY-YXKY-20250188). Written informed consent was obtained from the parents or legal guardians of all participants. For the present study, 2 mL peripheral venous blood was collected from each participant into tubes containing citrate or EDTA anticoagulants to perform molecular genetic screening for Y-chromosome material. Genomic DNA was extracted from whole blood using a commercial DNA extraction kit (Tellgen Biotechnology Co., Ltd., Shanghai, China) according to the manufacturer’s instructions and stored at −80 °C until further analysis.

### Detection of Y chromosome microdeletion genes

To identify both overt and cryptic Y chromosome material, molecular genetic analyses were performed using polymerase chain reaction (PCR)–based assays. Y chromosome microdeletion screening was conducted using a commercially available detection kit (Shanghai Tellgen Biotechnology Co., Ltd.). The assay protocol followed the manufacturer’s instructions, which are based on the EAA/EMQN best practice guidelines for the molecular diagnosis of Y-chromosomal microdeletions (19). Each DNA sample was subjected to two independent PCR reactions (Real-Time Quantitative PCR and conventional PCR) to ensure analytical reliability. Amplification was carried out on a real-time quantitative PCR platform under the following conditions: initial incubation at 50 °C for 2 min; denaturation at 95 °C for 5 min; followed by 38 cycles of denaturation at 95 °C for 15 s, annealing at 60 °C for 30 s, and extension at 72 °C for 30 s; with a final extension at 72 °C for 5 min. Fluorescence signals (FAM, VIC, ROX, and Cy5) were collected during the annealing phase at 60 °C, and results were interpreted based on amplification curves and threshold cycle values. In addition, conventional PCR assays were conducted to amplify six specific Y-chromosome loci, with the dystrophin gene (DMD) serving as an internal control. The resulting PCR products were separated by 1.5% agarose gel electrophoresis and visualized under ultraviolet illumination to confirm gene retention patterns. Primer sequences are listed in **Table 1**. PCR reactions were conducted using DreamTaq Green PCR Master Mix (2×; Thermo Scientific, USA). The amplification protocol consisted of an initial denaturation at 95 °C for 3 min, followed by 34 cycles of denaturation at 95 °C for 30 s, annealing at 55 °C for 30 s, and extension at 72 °C for 2 min, with a final extension at 72 °C for 5 min. PCR products were separated by 1.5% agarose gel electrophoresis and visualized under ultraviolet illumination. To minimize the risk of contamination with exogenous male DNA, all DNA extraction and PCR procedures were performed by female laboratory personnel under strict contamination-control conditions. For each assay, nuclease-free water and genomic DNA from a healthy 46, XX female were used as negative controls, while DNA from a 46, XY male served as the positive control. Furthermore, all samples identified as harboring Y-chromosome material were retested using an independent PCR reaction targeting different Y-specific loci. Only samples yielding consistent positive signals across both assays were included in the Y-positive cohort.

**Table 1:** Primer sequences of genes.

Primer	Sequences
PABY	5’-GCGCCTATAGTGCCAGCTAC-3’ 5’-TGAGGGTCAGGCTGCTATTT-3’
DYZ1	5’-TCTTGAGTGTGTGGCTTTCG-3’ 5’-CCGTTCACATCAATTCCTTG-3’
DYZ3	5’-TGTGGTATGTGCATTCATCTCA-3’ 5’-GAATGCGCACAACAAAAAGA-3’
DYS231	5’-GGGATTGCAGAGAGCAAAAG-3’ 5’-GCCGTGTGCTGGAGACTAAT-3’
SRY	5’-CTAAGTATCAGTGTGAAACGGG-3’ 5’-CCTTCCGACGAGGTCGATAC-3’
TPSY	5’-CTGACGAAGATGAAGACATGC-3’ 5’-AGTCCCCTGACGTCTCTGTT-3’
DMD	5’-GGATTGCAACAAACCAACAGTG-3’ 5’-GGATTGCAACAAACCAACAGTG-3’

### Clinical and endocrine evaluation following

For patients identified as carrying Y chromosome material, detailed clinical assessments were performed. Gonadal tumor occurrence was determined based on surgical and pathological findings in patients who underwent gonadectomy. Pubertal development was evaluated according to Tanner staging, with particular attention to spontaneous breast development and menstrual history. Pubertal development was clinically assessed by pediatric endocrinologists according to Tanner staging (B1–B5 for breast development and P1–P5 for pubic hair). Patients were considered to have initiated puberty if they had attained Tanner Stage B2 or higher. Serum levels of FSH, LH, and AMH were measured using standard immunoassays in the hospital’s certified clinical laboratory. Pelvic ultrasonography was performed to assess uterine and ovarian morphology, including uterine dimensions and ovarian volume, when visualization was possible. Hormone levels, uterine and ovarian volume were interpreted according to age-specific reference ranges (20, 21).

### Statistical analysis

Categorical variables were presented as frequencies and percentages. Descriptive statistical methods, including pie charts, bar charts, and scatter plots, were utilized to visualize the distribution and statistical results. Comparisons between patients with overt Y chromosome material and those with cryptic Y chromosome fragments were performed using the chi-square test or Fisher’s exact test, as appropriate. A two-sided p value < 0.05 was considered statistically significant. Statistical analyses were conducted using SPSS software (version 26.0; IBM Corp., Armonk, NY, USA).

## Results

### Chromosomal karyotype distribution

A total of 224 adolescent girls diagnosed with Turner syndrome, all younger than 18 years, were included in this study. The diagnosis was confirmed by peripheral blood karyotype analysis using standard cytogenetic techniques. The distribution of chromosomal karyotypes is summarized in **Figure 1b**; **Supplementary Tables S1**–**S3**. Monosomy 45,X was the most frequent karyotype, accounting for 35.27% of cases. Mosaic karyotypes, including 45,X/46,XX and 45,X/47,XXX, represented 12.95% of the cohort. Additional structural abnormalities and mosaic variants—such as ring X chromosomes, marker chromosomes, isochromosomes, and various deletions or derivative chromosomes—were also observed, reflecting the cytogenetic heterogeneity characteristic of TS.

### PCR-based Y chromosome detection

**Figure 2:**
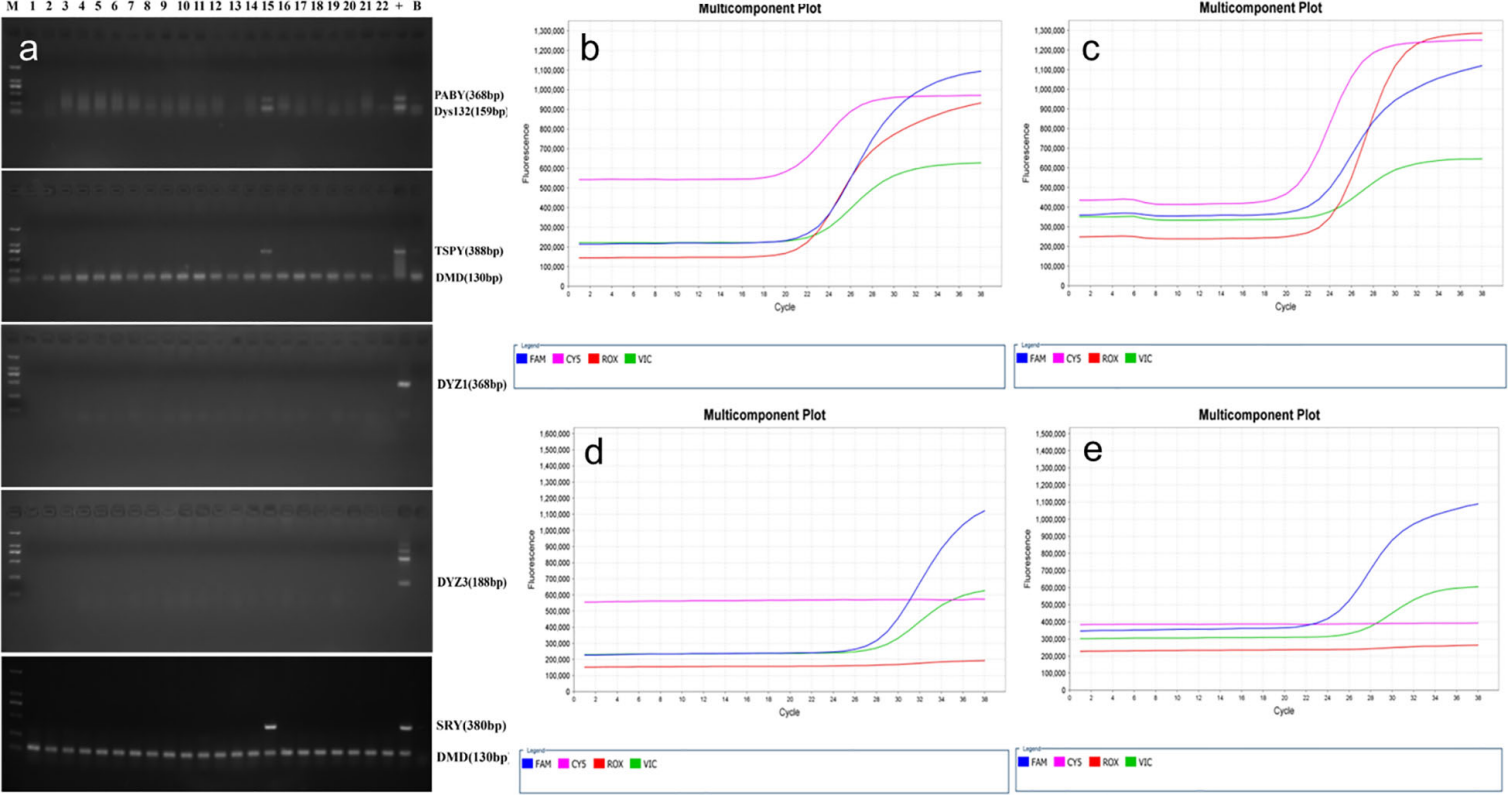
Molecular detection and characterization of Y-chromosome sequences in TS patients. **(a)** Representative agarose gel electrophoresis image for Y-specific gene screening in a subset of 224 TS patients. Lane 15 demonstrates a positive profile for PABY, DYS132, TSPY, and SRY genes. “+” and “–” denote the positive and negative controls, respectively. **(b–e)** Quantitative PCR (qPCR) multicomponent plots illustrating various Y-chromosome microdeletion patterns based on four fluorescence channels: FAM (reference genes SRY and ZFX/ZFY), VIC (sY84 and sY86 in the AZFa region), ROX (sY127 and sY134 in the AZFb region), and Cy5 (sY255 and sY254 in the AZFc region). Panels **(b, c)** display intact Y-chromosome sequences with robust amplification across all channels. Panels **(d, e)** show partial microdeletions, characterized by the absence of fluorescence signals in the ROX (AZFb) and Cy5 (AZFc) channels.

**Figure 3:**
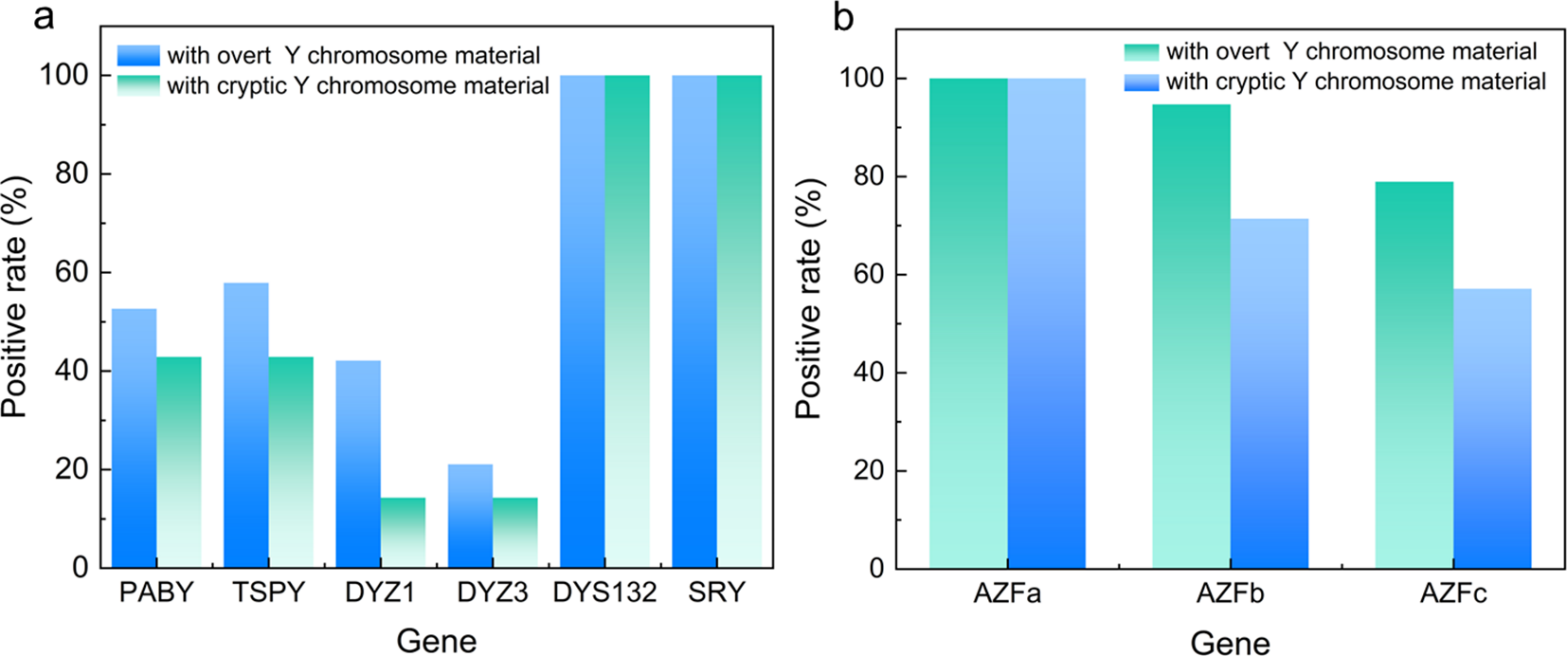
**(a)** In the 26 TS+Y patients, gel electrophoresis detection was performed, and the number of patients with specific Y chromosome genes is shown as a percentage of the total 26 TS+Y patients. **(b)** Q-PCR detection was performed using a Y chromosome deletion kit, and the number of patients with specific Y chromosome genes is shown as a percentage of the total 26 TS+Y patients.

Among the 224 TS patients, Y chromosome material was identified in 26 individuals (11.6%). Of these, 19 patients had overt Y chromosome material detectable by conventional karyotyping, whereas 7 patients harbored cryptic Y chromosome fragments that were identified only by molecular genetic analyses (**Figure 2**). This finding underscores the added diagnostic value of PCR-based assays in detecting low-level or cryptic Y chromosome materials that may be missed by conventional karyotyping. PCR analysis of Y chromosome–specific loci in the 26 TS+Y patients revealed heterogeneous gene retention patterns (**Figure 3a**; **Supplementary Table S1**). All patients tested positive for the SRY gene, while more than half also carried TSPY and PABY sequences. In contrast, the centromeric marker DYZ3 showed the lowest detection rate. Partial Y chromosome deletions were identified in 7 of the 26 TS+Y patients, whereas the remaining 19 patients showed no detectable deletions across the analyzed loci. Quantitative PCR amplification curves for representative Y chromosome genes are shown in **Figure 3b**; **Supplementary Table S2**.

### Gonadectomy and gonadal tumor findings

**Figure 4:**
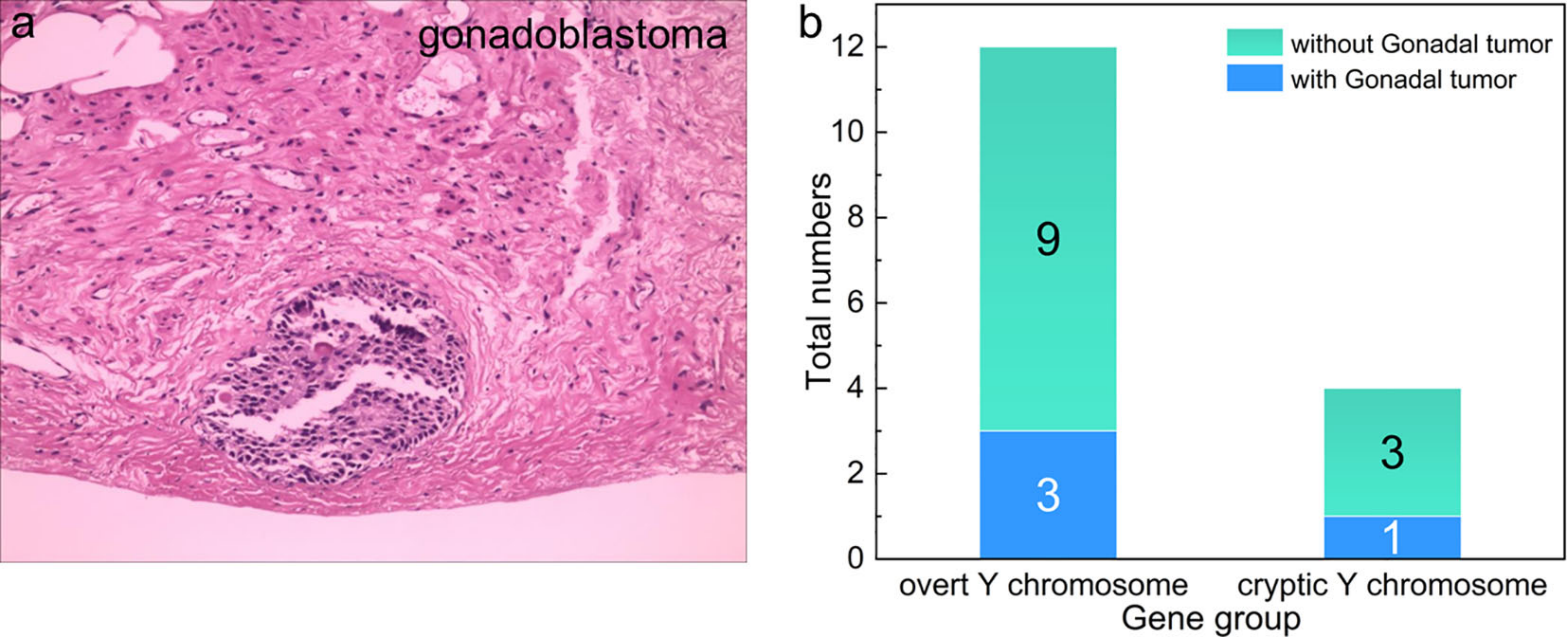
Histopathological and clinical characterization of gonadal tumors in TS+Y patients. **(a)** Representative histopathological section of a gonadoblastoma (200× magnification, HE staining). Post-fixation examination reveals characteristic nests of tumor cells distributed within the dense fibrous stroma. The neoplastic population comprises a majority of cells with hyperchromatic nuclei, inconspicuous nucleoli, and scant cytoplasm, alongside a minority of cells exhibiting clear cytoplasm. **(b)** Clinical distribution and tumor incidence among the 16 TS+Y patients who underwent bilateral prophylactic gonadectomy. The bar chart compares the occurrence of pathologically confirmed gonadal tumors between patients with overt (karyotypically visible) and cryptic (detected only by PCR) Y-chromosome material.

Sixteen of the 26 TS+Y patients underwent bilateral gonadectomy during the study period. Histopathological examination confirmed gonadal tumors in 4 of these 16 patients (25.0%), all of which were diagnosed as gonadol tumor. Representative histological features are shown in **Figure 4a**. Of the four tumor-positive cases, three occurred in patients with overt Y chromosome material and one in a patient with cryptic Y chromosome fragments (**Figure 4b**; **Supplementary Table S3**). Although a higher proportion of tumors was observed in patients with overt Y chromosome material, the difference between the two groups did not reach statistical significance. Preoperative pelvic ultrasonography did not identify definitive gonadal masses in any of the tumor-positive patients. Intraoperatively, the gonads were frequently described as fibrotic or streak-like, highlighting the limited sensitivity of imaging modalities for detecting neoplastic changes in dysgenetic gonadal tissue.

### Serum sex hormone profiles

**Figure 5:**
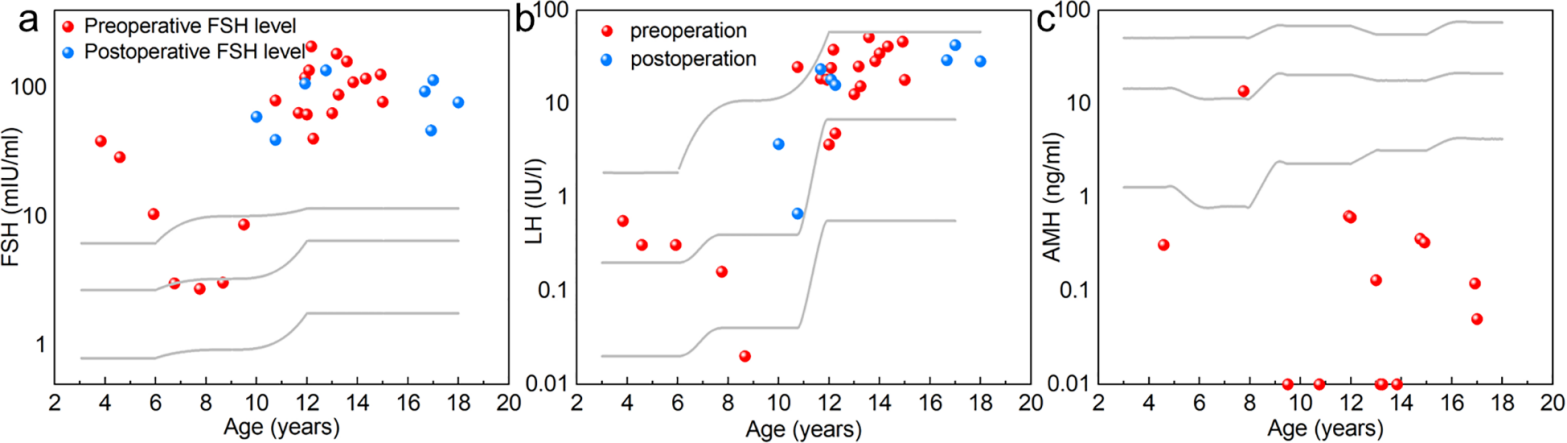
Distribution of serum gonadotropins and AMH levels in TS+Y patients relative to age-specific norms. **(a)** Preoperative (red dots) and postoperative (blue dots) serum FSH levels. Gray curves indicate the 2.5th, 50th, and 97.5th percentile reference ranges. **(b)** Preoperative (red dots) and postoperative (blue dots) serum LH levels. The three gray curves represent the 2.5th, 50th, and 97.5th percentiles of LH reference values for healthy girls. **(c)** Serum AMH concentrations in TS+Y patients (red dots). Most values fall significantly below the age-matched 50th percentile reference curve (middle gray line), indicating a severely diminished primordial follicle pool. In most panels, the age-dependent escalation of gonadotropins (LH and FSH) and the corresponding decline in AMH highlight the progression toward hypergonadotropic hypogonadism.

Serum gonadotropin measurements were available for 24 of the 26 TS+Y patients. Most patients exhibited elevated FSH levels relative to age-matched reference ranges, particularly among those who had entered puberty (**Figure 5a**; **Supplementary Table S4**). In contrast, prepubertal patients were more likely to demonstrate FSH values within the normal range. A similar pattern was observed for LH levels, with postpubertal patients showing markedly elevated concentrations (**Figure 5b**; **Supplementary Table S4**), consistent with hypergonadotropic hypogonadism. Serum AMH levels were measured in 15 TS+Y patients (**Figure 5c**; **Supplementary Table S5**). Only one patient— prepubertal and younger than 10 years—had AMH concentrations within the normal reference range. The remaining 14 patients exhibited AMH levels below the lower limit of normal, indicating markedly reduced ovarian reserve.

### Uterine and ovarian ultrasonographic findings

**Figure 6:**
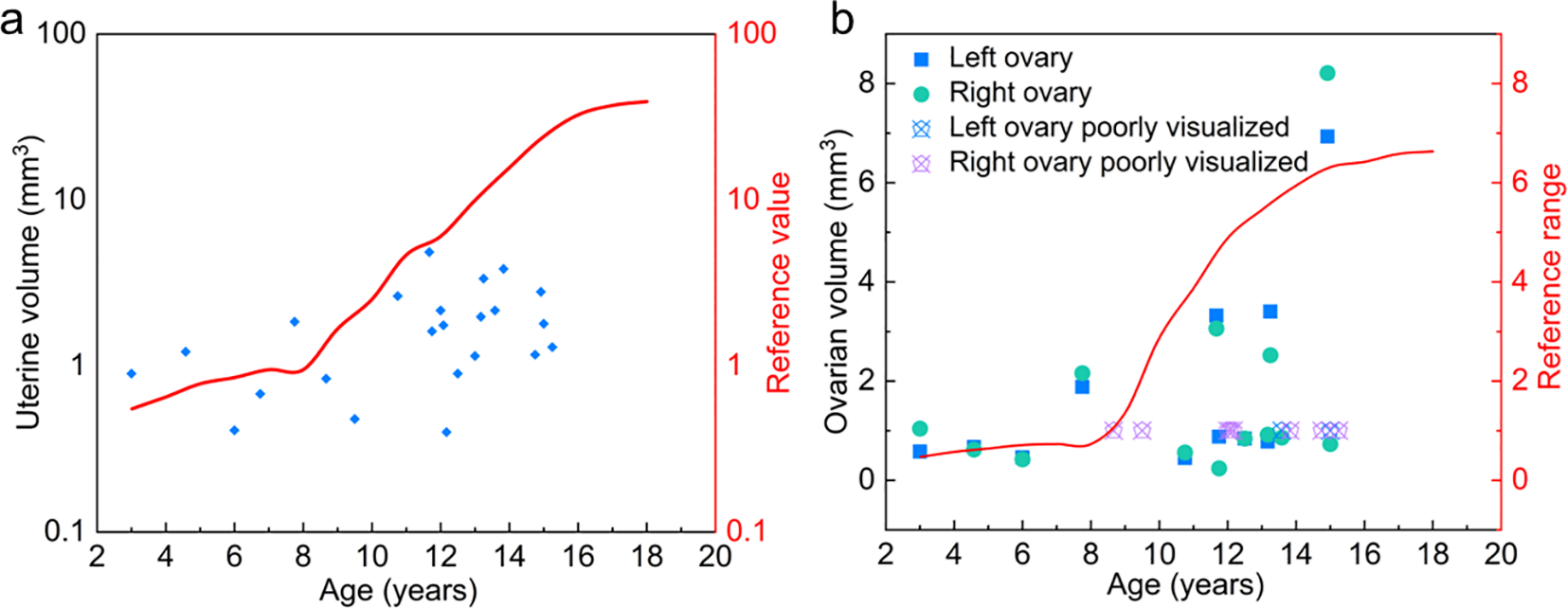
Pelvic ultrasonographic assessment of uterine and ovarian development in TS+Y patients. **(a)** Distribution of uterine volumes across different age groups. Blue dots represent individual measurements of TS+Y patients, while the red curve indicates the 50th percentile reference for healthy, age-matched girls. Uterine volume was calculated based on length, width, and endometrial thickness, with most patients exhibiting significant hypoplasia relative to normative data. **(b)** Individual ovarian volumes in TS+Y patients. Blue and green dots represent the left and right ovarian volumes, respectively, while the specialized symbols indicate cases where the ovaries were poorly visualized or presented as streak-like structures. The red curve denotes the 50th percentile reference for normal ovarian volume at each age. The consistent clustering of data points below the reference curves highlights the profound structural gonadal dysgenesis and limited pubertal progression in this population.

Pelvic ultrasonography data were available for 24 of the 26 TS+Y patients. Most patients demonstrated reduced uterine size compared with age-specific normative values (**Figure 6a**; **Supplementary Table S6**). Only three patients had uterine dimensions within the normal range, all of whom were prepubertal. Ovarian visualization was limited in a substantial proportion of patients; in 13 cases, one or both ovaries could not be clearly identified on ultrasound (**Figure 6b**; **Supplementary Table S6**). When visualized, most ovarian volumes were consistently below the 50th percentile for age-matched controls, suggesting ovarian hypoplasia or severe gonadal dysgenesis. Collectively, the endocrine and imaging findings indicate that most TS patients with Y chromosome material have severely compromised gonadal development and limited potential for spontaneous pubertal progression or fertility.

## Discussion

In the present study, the 11.6% prevalence of Y chromosome material identified among 224 patients reinforces the current consensus that molecular-based techniques significantly outperform conventional cytogenetics in detecting Y-derived sequences. This high detection rate, consistent with recent molecular findings, further confirms that conventional karyotyping lacks the requisite sensitivity for comprehensive screening. While international guidelines like the ACMG 2010 (22) appropriately designate conventional karyotyping as the gold standard for TS diagnosis, its inherent detection limit for low-level mosaicism presents a challenge for oncologic risk management. Our findings suggest that molecular techniques should not replace but rather complement conventional cytogenetics. By integrating PCR as an adjunctive screening tool, clinicians can achieve a higher sensitivity for detecting obvious Y-chromosome material, which our study proves can lead to gonadoblastoma even when undetected by the gold standard test Beyond mere detection, our integrated approach—combining molecular diagnostics with endocrine and imaging data—enables a more precise stratification of tumor risk and reproductive potential, addressing the clinical uncertainties highlighted earlier. This comprehensive evaluation clarifies the temporal window for potential intervention and informs the delicate balance between gonadectomy and fertility counseling. Collectively, our findings provide critical insights into the clinical trajectory of TS+Y patients. First, both overt and cryptic Y chromosome material confer a non-negligible risk of gonadoblastoma, necessitating vigilant screening regardless of the mosaicism level, particularly in individuals with a 45,X karyotype and signs of virilization. None of the 45,X individuals with Y-chromosome material in our cohort exhibited virilization at diagnosis, likely due to their young age—most were prepubertal or in early puberty, when androgen production is inactive. Additionally, this may also be attributed to the limitation of the limited sample size in our center; expanding the number of study subjects might reveal 45,X patients with virilization features. However, in our cohort, three patients with identifiable Y-chromosomal material presented with the phenotype of clitoromegaly. Second, the vast majority of TS+Y individuals exhibit severely compromised endocrine function and a diminished ovarian reserve. Finally, residual gonadal activity, when present, is observed primarily within a specific prepubertal or early pubertal window in our cohort. While the longevity of this activity remains to be fully elucidated through longitudinal studies, our findings suggest that fertility-preserving strategies or clinical interventions may benefit from individualized monitoring and early counseling.

The association between Y-chromosome material and gonadoblastoma in Turner syndrome (TS) is well established, though reported incidences in the literature fluctuate significantly—typically ranging from 3% to 15% —due to heterogeneities in patient age, selection criteria, and detection sensitivity (8–13). Notably, while the risk of malignant transformation remains relatively low at 3.5% (23), the precursor lesion—gonadoblastoma—presents a persistent threat. In our cohort, gonadal tumors were identified in 25.0% (4/16) of patients who underwent prophylactic gonadectomy. This prevalence is significantly higher than the ranges reported in recent large-scale studies, likely reflecting our inclusion of adolescents and the utilization of molecular techniques capable of identifying cryptic Y fragments that might otherwise be overlooked (9, 12). Intriguingly, while three tumor cases occurred in patients with karyotypically overt Y material, one tumor was detected in a patient with cryptic fragments identified solely by PCR. This finding, though limited by sample size, reinforces the critical concept that cryptic Y material is not clinically benign. Biologically, this risk is largely attributed to Y-linked genes, specifically TSPY within the GBY (Gonadoblastoma on the Y chromosome) region, which drives aberrant germ cell proliferation in dysgenetic gonads (24). With over half of our TS+Y patients harboring TSPY sequences, our data provide a molecular rationale for tumor development even in cytogenetically “negative” cases. Consequently, detecting cryptic Y material via PCR is not merely a diagnostic supplement but a clinical necessity for accurate oncological risk stratification.

A clinically critical observation in our cohort was the failure of preoperative pelvic ultrasonography to detect gonadal masses in any of the tumor-positive patients. Intraoperative findings and histopathological reviews frequently identified streak-like or fibrotic gonads containing only microscopic foci of gonadoblastoma. These findings underscore the inherent limitations of conventional imaging in monitoring dysgenetic gonads. Our data reinforce the consensus that normal sonographic findings are insufficient to exclude neoplastic transformation when Y-chromosome material is present (25). Given that undifferentiated gonadal tissue serves as a substrate for tumorigenesis (26), and that the risk of germ cell tumor (GCT) transformation peaks during puberty and early adulthood (27, 28), relying on imaging-based surveillance may lead to a dangerous delay in diagnosis. Therefore, we recommend PCR-based molecular analysis as a critical diagnostic supplement for all TS patients who are identified as Y-negative via conventional karyotyping, in order to achieve a more sensitive and comprehensive oncologic risk assessment.

Furthermore, detailed endocrine profiling revealed a distinct, age-dependent trajectory of gonadal exhaustion in TS+Y patients. The observed LH and FSH levels in our prepubertal cohort fell within the age-appropriate reference range; however, this reflects the physiological suppression of the hypothalamic-pituitary-gonadal (HPG) axis during childhood rather than active gonadal endocrine function. AMH levels, a robust surrogate for the primordial follicle pool, provided further evidence of this rapid depletion. A significant age-related decline in ovarian reserve was observed within the cohort. Among patients younger than 10 years, only one individual maintained an AMH level within the age-specific normal range, while the remainder exhibited values below the reference threshold. In contrast, for all patients aged 10 years or older, AMH levels were consistently and markedly below the age-matched reference ranges, signifying a severe depletion of the primordial follicle pool. This accelerated loss of ovarian reserve aligns with longitudinal observations in TS populations, where structural or numerical X-chromosome abnormalities drive a steep, early-childhood decline in AMH (29–31). These findings suggest that for the rare subset of TS+Y patients with residual gonadal function, the window for potential fertility-preserving interventions is exceptionally narrow and closes prematurely.

Pelvic ultrasonographic findings further corroborated the endocrine profiles. Ovarian visualization was frequently unsuccessful; when identifiable, gonadal volumes were significantly below age-matched norms. Crucially, even in the subset of patients with spontaneous breast development, ovarian morphology remained predominantly hypoplastic. This suggests that transient estrogen production may occasionally occur despite profound structural dysgenesis, representing a “remnant” function rather than sustained reproductive potential. Collectively, our multi-modal data indicate that spontaneous puberty and natural fertility are exceptional outcomes in TS+Y patients, typically confined to a fleeting window in early childhood or early adolescence.

The central clinical dilemma in TS+Y management remains the balance between mitigating malignancy risk and preserving residual gonadal function (16). Beyond the balance of oncologic risk and fertility potential, the management of TS patients with Y-chromosome material must also consider the ethical dimension of bodily integrity. Prophylactic surgery in asymptomatic minors involves the irreversible removal of gonadal tissue that may still possess endocrine function. In select prepubertal patients with detectable AMH levels and preserved gonadotropin profiles, fertility preservation (FP) strategies may be theoretically discussed within an ethically approved framework. However, ovarian tissue cryopreservation (OTC) is generally considered a relative contraindication for individuals harboring Y-chromosome material, given the significant risk of malignant transformation in the transplanted tissue (32, 33). While oocyte vitrification following *in vitro* maturation (IVM) may emerge as a potential alternative (34), it must be emphasized that IVM remains an experimental and non-established technique within this specific clinical context. The experimental nature of these procedures introduces significant psychosocial complexity into the clinical decision-making process (35, 36). Therefore, although fertility preservation is an important consideration, the timing of preventative surgery should be carefully optimized to minimize the risk of cancer, given the current limitations of diagnostic monitoring and the unpredictability of malignant transformation. Our data suggest that for the vast majority of patients, the window of meaningful gonadal activity is narrow and often closes before gonadectomy is traditionally performed. Consequently, early prophylactic gonadectomy is justified for most, especially upon the onset of biochemical or imaging evidence of gonadal failure. However, our findings also advocate for a nuanced, individualized approach.

Several limitations of this study warrant consideration. First, the retrospective, single-center design may introduce selection bias, potentially constraining the generalizability of the results. Second, the relatively small sample size—particularly of those undergoing surgery—limits the statistical power for robust subgroup analyses comparing overt versus cryptic Y-chromosome material. While the difference in tumor incidence between these groups did not reach statistical significance (*P* > 0.05), this likely reflects the sample size rather than a parity of risk. Finally, genetic analysis was restricted to peripheral blood. Since Y-chromosome mosaicism can exhibit tissue-specific patterns, the results may not fully reflect the genetic status of other tissues, such as the gonads. From a safety perspective, any detectable Y-derived sequence must be managed as a potential oncological risk factor. Consequently, large-scale, prospective multicenter studies utilizing standardized molecular diagnostics and longitudinal profiling are essential to refine risk stratification and establish evidence-based management algorithms for this high-risk population.

## Conclusion

This integrated single-center study demonstrates that Y-chromosome material is present in a substantial subset of patients with Turner syndrome (TS) and confers a significant risk of gonadoblastoma, regardless of whether the Y sequences are cytogenetically overt or molecularly cryptic. Importantly, our data reveal that normal preoperative imaging fails to reliably exclude gonadal neoplasia, highlighting the inadequacy of ultrasonography as a standalone tool for oncologic risk stratification in this high-risk population. Beyond oncological concerns, our integrated endocrine and imaging analyses characterize the profound gonadal dysgenesis prevalent in TS+Y patients, typically manifested as hypergonadotropic hypogonadism, depleted AMH levels, and uterine hypoplasia. These findings suggest that meaningful reproductive potential is rare and, where it exists, remains uncertain and confined to a narrow prepubertal window.

Accordingly, these findings support early prophylactic gonadectomy as a robust risk-reducing strategy for the majority of TS patients with Y-chromosome material. However, management must remain individualized; in carefully selected prepubertal patients with biochemical evidence of residual gonadal activity, fertility preservation may be explored within a multidisciplinary ethical framework. Future prospective multicenter studies are warranted to refine risk stratification and optimize clinical decision-making in this heterogeneous population.

## Data Availability

The original contributions presented in the study are included in the article/**Supplementary Material**. Further inquiries can be directed to the corresponding authors.
