# Supplementary material for: Balancing oncologic risk and fertility potential: a single-center study on Turner syndrome patients with Y chromosome material
Source: Front Endocrinol (Lausanne). 2026 May 5;17:1793595. doi: 10.3389/fendo.2026.1793595 (PMC13184723; doi:10.3389/fendo.2026.1793595)
Supplement: Supplementary file 1 [file DataSheet1.docx]

**Supplementary materials**

**Balancing Oncologic Risk and Fertility Potential: A Single-Center Study on Turner Syndrome Patients with Y Chromosome Material**

**Xia Shuai^1^, Zhibing Guo^1^, Dongguang Zhang^1^, Hui Huang^1^, Ka Chen^1^, Yu Yang^1^*****, Qinghua Hu^1^***

^1^ *Development and Genetics Key Laboratory*, *Jiangxi Provincial Children's Hospital*, *Nanchang Medical College Affiliated Children's Hospital, Nanchang 330031, People’s Republic of China*

*Corresponding email: *[yangyu5168@126.com](mailto:yangyu5168@126.com)*; *[huqinghua2005tb@stu.xjtu.edu.cn](mailto:huqinghua2005tb@stu.xjtu.edu.cn)*

**Table S1. Y chromosome marker detection results in 26 patients**

| **ID** | **Karyotype** | **Diagnosis Age** | **PABY** | **TSPY** | **DYS132** | **DYZ1** | **DYZ3** | **SRY** | **DMD** |
| --- | --- | --- | --- | --- | --- | --- | --- | --- | --- |
| 1 | 45,X[41]/46,XY[9] | 14y8m | + | + | + | - | + | + | + |
| 2 | 45,X[31]/46,XY[19] | 14y11m | - | - | + | - | - | + | + |
| 3 | 45,X[12]/46,XY[30] | 14y4m | + | + | + | - | - | + | + |
| 4 | 45,X[92]/46,XY[8] | 6y | + | - | + | - | - | + | + |
| 5 | 45,X/46,XY | 2y9m | + | - | + | - | - | + | + |
| 6 | 45,X[5]/46,XY[25] | 11y | + | - | + | + | + | + | + |
| 7 | 45,X[4]/46,XY[46] | 3y6m | + | + | + | + | - | + | + |
| 8 | 45,X[11]/46,XY[22] | 13y3m | - | - | + | + | - | + | + |
| 9 | 45,X[27]/46,XY[13] | 7y | - | + | + | - | - | + | + |
| 10 | 45,X[6]/46,XY[44] | 13y2m | - | + | + | + | - | + | + |
| 11 | 45,X[23]/46,XY[27] | 13y7m | - | + | + | - | - | + | + |
| 12 | 45,X[9]/46,XY[91] | 10y | - | + | + | + | + | + | + |
| 13 | 45,X[9]/46,XY[41] | 13y10m | + | - | + | - | - | + | + |
| 14 | 45,X[27]/46,XY[23] | 12y2m | - | + | + | + | - | + | + |
| 15 | 45,X[40]/47,XYY[20] | 14y7m | - | - | + | - | - | + | + |
| 16 | 45,X[22]/46,X,r(X)[38]/46,X,idic(Y)[7]/47,X,+2mar[13] | 12y | + | + | + | - | + | + | + |
| 17 | 45,X,der(18;Y) | 4y9m | + | + | + | - | - | + | + |
| 18 | 45,X[44]/46,XY[4]/47,XXY[3]/46,X,der(Y)add(Y)(p11)[55] | 12y1m | + | - | + | + | - | + | + |
| 19 | 45,X/46,X,i(Y)/47,XY,+i(Y) | 8y | - | + | + | + | - | + | + |
| 20 | 45,X | 1y10m | - | + | + | - | - | + | + |
| 21 | 45,X | 6y11m | - | - | + | - | - | + | + |
| 22 | 45,X[12]/46,X,+mar[78] | 5y7m | + | + | + | - | + | + | + |
| 23 | 45,X[37]/46,X,+mar[13] | 12y3m | + | + | + | - | - | + | + |
| 24 | 45,X[19]/46,X,+mar[21] | 11y8m | - | - | + | + | - | + | + |
| 25 | 45,X[56]/46,X,+mar[4] | 9y | + | - | + | - | - | + | + |
| 26 | 45,X[41]/46,X,+mar[9] | 11y8m | - | - | + | - | - | + | + |
| **Total** | **26** | **10y±4y** | **13** | **14** | **26** | **9** | **5** | **26** | 26 |

**Table notes:**

Karyotypes are presented as reported; numbers in brackets indicate the number of cells with that karyotype in conventional cytogenetics (where available).

**PABY:**pseudoautosomal boundary region, Y-linked, marker indicates presence of Yp material.

**TSPY:**Testis specific protein ,Y-linked, a marker for the Y chromosome short arm.

**DYS132:**A sequence-tagged site (STS) on the Y chromosome long arm (Yq).

**DYZ1:**A repetitive satellite DNA sequence on Yq12, often used as a marker for the heterochromatic region.

**DYZ3:**Alphoid satellite DNA at the Y chromosome centromere.

**SRY:**Sex-determining region Y gene, located on Yp11.3.

**DMD:**Dystrophin gene (X-linked), included as an internal control or to assess X chromosome integrity.

**"+"** indicates the marker was detected (positive amplification); **"-"** indicates the marker was not detected (negative).

**Mean age at diagnosis:** 10.03 years (10y0m), SD: 4.04 years (4y0m),n=26.

**Table S2. Karyotypes and Y chromosome microdeletion test results of the 26 patients**

| **ID** | **Karyotype** | **AZFa (sY84+sY86)** | **AZFb (sY127)** | **AZFb (sY134)** | **AZFc (sY254+sY255)** |
| --- | --- | --- | --- | --- | --- |
| 1 | 45,X[41]/46,XY[9] | + | + | + | - |
| 2 | 45,X[31]/46,XY[19] | + | + | + | - |
| 3 | 45,X[12]/46,XY[30] | + | + | - | - |
| 4 | 45,X[92]/46,XY[8] | + | - | - | - |
| 5 | 45,X/46,XY | + | + | + | + |
| 6 | 45,X[5]/46,XY[25] | + | + | + | + |
| 7 | 45,X[4]/46,XY[46] | + | + | + | + |
| 8 | 45,X[11]/46,XY[22] | + | + | + | + |
| 9 | 45,X[27]/46,XY[13] | + | + | + | + |
| 10 | 45,X[6]/46,XY[44] | + | + | + | + |
| 11 | 45,X[23]/46,XY[27] | + | + | + | + |
| 12 | 45,X[9]/46,XY[91] | + | + | + | + |
| 13 | 45,X[9]/46,XY[41] | + | + | + | + |
| 14 | 45,X[27]/46,XY[23] | + | + | + | + |
| 15 | 45,X[40]/47,XYY[20] | + | + | + | + |
| 16 | 45,X[22]/46,X,r(X)[38]/46,X,idic(Y)[7]/47,X,+2mar[13] | + | + | + | + |
| 17 | 45,X,der(18;Y) | + | + | + | + |
| 18 | 45,X[44]/46,XY[4]/47,XXY[3]/46,X,der(Y)add(Y)(p11)[55] | + | + | + | + |
| 19 | 45,X/46,X,i(Y)/47,XY,+i(Y) | + | + | + | + |
| 20 | 45,X | + | + | + | - |
| 21 | 45,X | + | + | + | + |
| 22 | 45,X[12]/46,X,+mar[78] | + | - | - | - |
| 23 | 45,X[37]/46,X,+mar[13] | + | - | - | - |
| 24 | 45,X[19]/46,X,+mar[21] | + | + | + | + |
| 25 | 45,X[56]/46,X,+mar[4] | + | + | + | + |
| 26 | 45,X[41]/46,X,+mar[9] | + | + | + | + |
| **Positive cases (n)** | **26** | **26** | **23** | **22** | **19** |

**Table notes:**

**AZFa, AZFb, and AZFc** refer to three independent regions within the azoospermia factor (AZF) locus on the long arm of the Y chromosome. The specific sequence-tagged sites (STS) tested for deletions were: AZFa (sY84, sY86), AZFb (sY127, sY134), and AZFc (sY254, sY255). Two internal control genes were also included: the sex-determining region of the Y chromosome (SRY) and the zinc finger protein genes ZFX/ZFY.

**"+"** indicates that the corresponding STS marker showed a clear S-shaped amplification curve with a Ct value < 32, and the ZFX/ZFY internal control also showed a clear S-shaped amplification curve with a Ct value < 32.

**"–"** indicates that the corresponding STS marker showed no clear S-shaped amplification curve or had a Ct value ≥ 32, while the ZFX/ZFY internal control showed a clear S-shaped amplification curve with a Ct value < 32.

ID numbers correspond to those in Supplementary Table 1(Y chromosome marker detection ).

Deletions of a single STS marker in AZFa or AZFb are very rare; such occurrences in this study may be attributable to polymorphisms at the 5' end of the primer binding sites. sY254 and sY255 are located within the DAZ gene, which has four copies on the Y chromosome; therefore, deletion of a single sY254 or sY255 site is theoretically impossible.

**Table S3. Intraoperative and gonadal pathology findings in patients with Y chromosome anomalies**

| **ID** | **Karyotype** | **Surgery Age** | **Intraoperative Findings** | **Gonadal Pathology Findings** |
| --- | --- | --- | --- | --- |
| 1 | 45,X[41]/46,XY[9] | 15y3m | A hypoplastic uterus, bilateral tubal structures, and bilateral streak gonads | Left:Ovarian stroma was present, with no follicles observed. Focal calcifications, abundant fibrous tissue, and vascular structures were noted. Right:Ovarian stroma was present, with no follicles observed. Focal calcifications, abundant fibrous tissue, and vascular structures were noted. |
| 2 | 45,X[31]/46,XY[19] | 15y4m | A hypoplastic uterus, bilateral tubal structures, and bilateral streak gonads | Left:Ovarian stroma and a serous cyst were observed. Right:Ovary-like stroma was present.Diagnosis:Gonadal sex cord-stromal tumor. |
| 3 | 45,X[12]/46,XY[30] | 14y9m | A hypoplastic uterus, bilateral tubal structures, and bilateral streak gonads | The left ovary exhibited dysplasia with granulosa cell tumor, while the right ovary showed dysplasia. |
| 4 | 45,X[92]/46,XY[8] | 6y | A hypoplastic uterus, bilateral tubal structures, and bilateral streak gonads | Bilateral sections revealed abundant fibrous and vascular tissue, with sparse ovarian stroma-like components and a few tubule-like structures. |
| 6 | 45,X[5]/46,XY[25] | 12y | A hypoplastic uterus, bilateral tubal structures, and bilateral streak gonads; a soybean-sized mass was found adjacent to the left fallopian tube. | A left paraovarian cyst was identified, along with bilateral dysplastic gonads, both of which contained ovarian stroma. |
| 8 | 45,X[11]/46,XY[22] | 13y3m | The uterus and fallopian tubes were present, along with bilateral streak gonads. Additionally, a yellow mass was observed in the right pelvic cavity. | Bilateral ovarian stroma-like structures and glandular tubular structures were observed, along with fibrous tissue and blood vessels. A pelvic mass composed of fibrous tissue with calcification was also noted. |
| 9 | 45,X[27]/46,XY[13] | 7y9m | A uterus-like structure and left tubo-ovarian-like structure were observed, along with a right streak gonad. | 1. Mesonephric duct cyst 2. Clitoral hypertrophy |
| 11 | 45,X[23]/46,XY[27] | 13y10m | A hypoplastic uterus, bilateral tubal structures, and bilateral streak gonads | Bilateral streak gonads were observed. The surface of each gonad was covered by a thin layer of stroma, beneath which were smooth muscle, blood vessels, hyperplastic stromal cells, and a few dilated glands, in that order. |
| 12 | 45,X[9]/46,XY[91] | 13y | A hypoplastic uterus, bilateral tubal structures, and bilateral streak gonads | Left:Ovarian stroma with occasional follicular structures, fibrous tissue, and adipose tissue. Right:Ovarian stroma with occasional follicular structures, fibrous tissue, and vascular structures. |
| 14 | 45,X[27]/46,XY[23] | 12y2m | A hypoplastic uterus, bilateral tubal structures, and bilateral streak gonads | Gonad: Streak-like tissue with ovarian stroma-like structures and stromal cell hyperplasia. |
| 17 | 45,X,der(18;Y) | 7y | A hypoplastic uterus, bilateral tubal structures, and bilateral streak gonads | Bilateral fibrous tissue, blood vessels, and ovarian stroma-like structures were observed. Additionally, a gonadoblastoma was identified in the left gonad. |
| 18 | 45,X[44]/46,XY[4]/47,XXY[3]/46,X,der(Y)add(Y)(p11)[55] | 12y6m | A pea-sized dark brown mass was noted on the dorsal side of the clitoris. The pelvic cavity revealed a hypoplastic uterus, bilateral tubal structures, and bilateral streak gonads. | Gonads (bilateral):Ovary-like stroma, glandular tubular structures, fibrous tissue, and blood vessels. Clitoral prepuce (compound nevus):Proliferative nevus cells were observed in the epidermis and dermis. Immunohistochemistry:CR (+), vimentin (+), SMA (+), CK (+), CD117 (–). |
| 21 | 45,X | 10y9m | The uterine body was absent. Bilateral tubal structures were present but poorly developed, along with bilateral streak gonads. | Left:Ovarian stroma with a few glands. Right:Ovarian stroma with sparse glands and tubal-like structures. |
| 22 | 45,X[12]/46,X,+mar[78] | 8y7m | A hypoplastic uterus, bilateral tubal structures, and bilateral streak gonads | Left:Ovarian stroma, testicular seminiferous tubule-like structures, fibrous tissue, and blood vessels. Right:Fibrous tissue, blood vessels, tubal-like structures, and ovarian stroma. |
| 23 | 45,X[37]/46,X,+mar[13] | 12y5m | A left streak gonad and a right gonad with dysplastic fallopian fimbriae were observed. | Left gonad:Mostly fibrous tissue, blood vessels, and adipose tissue, with a focal vas deferens-like structure and sparse ovarian stroma. Right gonad:Testicular seminiferous tubules, vas deferens, and tubal-like structures. Adipose tissue and tubular structures. Clitoris:Irregularly dilated blood vessels and fibrous tissue hyperplasia with hyaline degeneration. |
| 24 | 45,X[19]/46,X,+mar[21] | 11y8m | A left gonadoblastoma and a right adrenal tumor were identified. | Left gonad:Fibrous tissue, blood vessels, nerve fibers, and glandular tubular structures, with gonadoblastoma. Right gonad:Vas deferens, fibrous tissue, blood vessels, nerve fibers, and a small nest of cells with abundant cytoplasm, along with stromal cell hyperplasia. Right adrenal gland:Ganglioneuroblastoma. |

**Table note:**

Patients are numbered according to the original study cohort. ID numbers correspond to those in Supplementary Table 1(Y chromosome marker detection ).

Mean age at surgery: 11.64 years (11y8m), SD: 2.89 years (2y11m), n=16.

**Table S4. Preoperative and postoperative hormone levels in 26 patients**

| **ID** | **Surgical Intervention** | **Preoperative Age** | **Preoperative** | | | | **Postoperative Age** | **Postoperative** | | | |
| --- | --- | --- | --- | --- | --- | --- | --- | --- | --- | --- | --- |
|  |  |  | FSH  (IU/L) | LH  (IU/L) | E2  (pg/mL) | T  (ng/dL) |  | FSH  (IU/L) | LH  (IU/L) | E2  (pg/mL) | T  (ng/dL) |
| 1 | Yes | 15y | 77.94 | 18.07 | 17.23 | 51.21 | 18y | 77.24 | 28.56 | <11.80 | 23.29 |
| 2 | Yes | 14y11m | 127.08 | 46.34 | <15.0 | 29.69 | 17y | 115.19 | 42.5 | <11.80 | 13.77 |
| 3 | Yes | 14y4m | 118.2 | 41.18 | <11.80 | 42.28 | 16y8m | 94.12 | 29.38 | <11.80 | 16.84 |
| 4 | Yes | 5y11m | 10.49 | 0.31 | <11.80 | 18.54 | — | — | — | — | — |
| 5 | No | — | — | — | — | — | — | — | — | — | — |
| 6 | Yes | 12y | 62.29 | 3.68 | <11.80 | 7.38 | — | — | — | — | — |
| 7 | No | 9y6m* | 8.7 | <0.07 | 28.88 | — | 10y | 59.71 | 3.72 | 33.07 | — |
| 8 | Yes | 13y3m | 88.75 | 15.46 | <11.80 | 11.17 | — | — | — | — | — |
| 9 | Yes | 7y9m | 2.76 | 0.16 | <15.0 | <10.0 | — | — | — | — | — |
| 10 | No | 13y2m* | 184.35 | 25.16 | 0.74 | 6 | — | — | — | — | — |
| 11 | Yes | 13y7m | 160.59 | 51.58 | <15.0 | 33.44 | — | — | — | — | — |
| 12 | Yes | 13y | 63.69 | 12.73 | <15.0 | 17.09 | — | — | — | — | — |
| 13 | No | 13y10m* | 110.89 | 28.7 | 12.81 | — | — | — | — | — | — |
| 14 | Yes | 12y2m | 210.35 | 37.97 | 7.52 | <10.0 | — | — | — | — | — |
| 15 | No | 4y7m* | 29.02 | 0.31 | 11.91 | 7.37 | — | — | — | — | — |
| 16 | No | 11y8m* | 63.96 | 18.77 | 21.87 | 117.9 | — | — | — | — | — |
| 17 | Yes | 6y9m | 3.04 | 0 | <1.80 | <7.0 | 10y9m | 39.52 | 0.67 | 6.07 | <10.0 |
| 18 | Yes | 12y1m | 137.17 | 24.31 | <11.80 | 26.82 | 12y9m | 137.06 | 18.16 | — | 12.55 |
| 19 | No | 14y11m* | 179.15 | 34.68 | <11.80 | 23.46 | — | — | — | — | — |
| 20 | No | 3y10m* | 38.66 | 0.56 | <1.80 | <7.0 | — | — | — | — | — |
| 21 | Yes | 10y9m | 79.99 | 24.77 | <15.0 | 13.98 | — | — | — | — | — |
| 22 | Yes | 8y6m | 3.08 | 0.02 | <11.80 | — | — | — | — | — | — |
| 23 | Yes | 12y3m | 40.45 | 4.83 | <11.80 | 24.36 | 16y11m | 46.81 | 15.95 | 39.05 | <7.0 |
| 24 | Yes | 11y8m | 120.55 | 18.16 | <1.80 | 15.65 | 11y11m | 108.15 | 23.55 | 3.3 | — |
| 25 | No | — | — | — | — | — | — | — | — | — | — |
| 26 | No | 11y8m | 60.27 | 12.96 | — | — | — | — | — | — | — |

**Table notes:**

**FSH:**Follicle-stimulating hormone;**LH:**Luteinizing hormone;**E2:**Estradiol;**T:**Testosterone.

**Units:** FSH and LH are reported in IU/L; E2 in pg/mL; T in ng/dL.

**“—”** indicate that hormone levels data were not measured.

*For patients who did not undergo surgery, "Preoperative Age" refers to the age at initial hormone evaluation.

Patient IDs correspond to those in Supplementary Table 1(Y chromosome marker detection ).

**Table S5.AMH and Turner staging in 26 patients**

| **ID** | **Age at Assessment** | **AMH (ng/mL)** | **Tanner Staging** | **Clinical features** |
| --- | --- | --- | --- | --- |
| 1 | Not measured | — | — | — |
| 2 | 17y | 0.05 | B1, PH1 | Height < -3SD, facial multiple nevi, low hairline |
| 3 | 14y9m | 0.36 | — | Height < -2SD |
| 4 | 5y11m | — | B1, PH1 | Height < -3SD, bilateral ptosis |
| 5 | — | — | — | — |
| 6 | 12y | 0.61 | B1, PH1 | Height < -3SD, facial multiple nevi, cubitus valgus |
| 7 | 9y6m | <0.10 | B1, PH1 | Height < -2SD |
| 8 | 13y3m | <0.10 | B1, PH1 | Height < -2SD |
| 9 | 7y9m | 13.7 | B1, PH1 | Height < -2SD, clitoromegaly |
| 10 | 13y2m | <0.10 | B1, PH1 | Height < -3SD, facial multiple nevi, low hairline, short neck, simian crease |
| 11 | 13y10m | <0.10 | B1, PH1 | Height < -3SD |
| 12 | 13y | 0.13 | B1, PH1 | Height < -2SD |
| 13 | 13y10m | — | B3, PH1 | Height < -3SD, facial multiple nevi, cubitus valgus |
| 14 | 12y2m | — | B1, PH1 | Height < -3SD, facial multiple nevi, widely spaced nipples |
| 15 | 4y7m | 0.31 | B1, PH1 | Height < -3SD, right hand polydactyly, right ptosis, epicanthal folds, webbed neck, pectus excavatum |
| 16 | 11y8m | — | B1, PH1 | Height < -3SD, facial multiple nevi, low-set ears, webbed neck, widely spaced nipples, clitoromegaly |
| 17 | 6y9m | — | B1, PH1 | Height < -3SD, cubitus valgus |
| 18 | 12y1m | — | B1, PH1 | Height < -3SD, facial multiple nevi, webbed neck |
| 19 | 14y11m | 0.33 | B4, PH1 | Height < -3SD, widely spaced nipples, shield chest, cubitus valgus |
| 20 | 3y10m | — | B1, PH1 | Height < -3SD, low-set ears, shield chest |
| 21 | 10y9m | <0.10 | B1, PH1 | Height < -3SD, facial multiple nevi |
| 22 | 8y6m | — | B1, PH1 | Height < -3SD |
| 23 | 16y11m | 0.12 | B1, PH1 | Height < -3SD, facial multiple nevi, high-arched palate, webbed neck, widely spaced nipples |
| 24 | 11y11m | 0.63 | B2*, PH1 | Height < -3SD, facial multiple nevi, shield chest, widely spaced nipples |
| 25 | — | — | — | — |
| 26 | 11y8m | 1.21 | B1, PH1 | Height < -2SD, widely spaced nipples,clitoromegaly |

**Table notes:**

**AMH:**Anti-Müllerian hormone, reported in ng/mL.

**Turner staging:**B = Breast development (B1–B5); PH = Pubic hair (PH1–PH5).

**“—”** indicate that data were not available or not measured.

"**Not measured**" indicates that the patient did not undergo assessment at the specified time point.

Patient IDs correspond to those in Supplementary Table1(Y chromosome marker detection ).

**“*”** indicate spontaneous breast development occurred without any medical intervention.

**Table S6. Uterine and ovarian volumes before and after surgical intervention**

| **ID** | **Surgical Intervention** | **Preoperative Age** | **Preoperative Uterine Volume**  **(mL)** | **Preoperative Left Ovary Volume**  **(mL)** | **Preoperative Right Ovary Volume (mL)** | **Postoperative Age** | **Postoperative Uterine Volume**  **(mL)** |
| --- | --- | --- | --- | --- | --- | --- | --- |
| 1 | Yes | 15y | 1.3 | Not visualized | Not visualized | 18y | 12.7 |
| 2 | Yes | 14y11m | 1.8 | Not visualized | 0.73 | 17y | 8.17 |
| 3 | Yes | 14y4m | 1.17 | Not visualized | Not visualized | 16y | 1.66 |
| 4 | Yes | 5y11m | 0.68 | Not visualized | Not visualized | 10y | 0.92 |
| 5 | No | — | — | — | — | — | — |
| 6 | Yes | 12y | 2.16 | Not visualized | Not visualized | — | — |
| 7 | No | 9y6m | 0.48 | Not visualized | Not visualized | — | — |
| 8 | Yes | 13y3m | 3.36 | 3.4 | 2.52 | — | — |
| 9 | Yes | 7y9m | 1.85 | 1.88 | 2.16 | — | — |
| 10 | No | 13y2m | 1.98 | 0.78 | 0.92 | — | — |
| 11 | Yes | 13y7m | 2.16 | Not visualized | 0.86 | — | — |
| 12 | Yes | 13y | 1.15 | Not visualized | Not visualized | — | — |
| 13 | No | 13y10m | 3.85 | Not visualized | Not visualized | — | — |
| 14 | Yes | 12y2m | 0.4 | Not visualized | Not visualized | 13y | 0.67 |
| 15 | No | 4y7m | 1.22 | 0.67 | 0.62 | — | — |
| 16 | No | 11y8m | 4.86 | 3.32 | 3.06 | — | — |
| 17 | Yes | 6y9m | 0.41 | 0.46 | 0.42 | 10y | 0.72 |
| 18 | Yes | 12y1m | 1.76 | Not visualized | Not visualized | 12y | 2.35 |
| 19 | No | 14y11m | 2.8 | 6.93 | 8.21 | — | — |
| 20 | No | 3y10m | 0.9 | 0.84 | 0.84 | 4y | 0.34 |
| 21 | Yes | 10y9m | 2.64 | 0.45 | 0.56 | — | — |
| 22 | Yes | 8y6m | 0.84 | Not visualized | Not visualized | 14y | 2.42 |
| 23 | Yes | 12y3m | 0.9 | 0.58 | 1.04 | 17y | 8.32 |
| 24 | Yes | 11y8m | 1.87 | Not visualized | Not visualized | 12y | 0.62 |
| 25 | No | — | — | — | — | — | — |
| 26 | No | 11y8m | 1.62 | 0.88 | 0.24 | — | — |

**Table notes:**

Organ volumes are expressed in milliliters (mL).

"Not visualized" indicates that the structure could not be identified on ultrasound examination.

**“—”** indicate that postoperative data were not available or not measured.

For patients who did not undergo surgery, "Preoperative Age" refers to the age at initial ultrasound evaluation.

Patient IDs correspond to those in Supplementary Table 1(Y chromosome marker detection ).
